# Effect of a moderate-intensity comprehensive exercise program on body composition, muscle strength, and physical performance in elderly females with sarcopenia

**DOI:** 10.1016/j.heliyon.2023.e18951

**Published:** 2023-08-06

**Authors:** Bo-yuan Chen, Yuan-zhe Chen, So-hee Shin, Chun-yang Jie, Zhi-liang Chang, Hui Ding, Hong Yang

**Affiliations:** a Department of Physical Education, Henan University of Science and Technology, Luoyang, 471023, China; b School of Sport and Exercise Science, University of Ulsan, 93 Daehak-ro, Nam-gu, Ulsan, 44610, South Korea

**Keywords:** arcopenia, Elderly females, Tai-chi, Elastic bands exercise, Physical performance

## Abstract

**Objective:**

This study aimed at examining an eight-week moderate-intensity comprehensive exercise training program on the parameters of sarcopenia in elderly females.

**Methods:**

A total of 49 community-dwelling elderly females with sarcopenia (65.5 ± 2.5) were assigned randomly to an experiment group (EG, n = 25) and a control group (CG, n = 24). In the EG, an eight-week comprehensive exercise training program was implemented, in 1 h, 3 times per week, a total of 24 sessions. The CG only received health public education per two weeks, a total of 4 times. Subsequently, the differences between the two groups were tested through two-way repeated ANOVA.

**Results:**

ASM, SMM, and SMI in the EG were significantly improved by 0.26 kg, 0.18 kg, and 0.10 kg/m^2^, respectively. Group-by-time interactions were significantly different on the ASM [F (1,47) = 6.25, η^2^ = 0.12] and SMI [F (1,47) = 6.77, η^2^ = 0.13]. Muscle strength was improved 0.8 kg in the EG. Significant group-by-time interaction differences were reported in the handgrip strength [F (1,47) = 6.8, η^2^ = 0.13] after the eight-week intervention. Compared with the baseline, gait speed was improved a 0.05 m/s and 5-time chair stand was decreased a 0.27 s in the EG. Group-by-time interactions were significantly different in 5-time chair stand [F (1, 47) = 6.35, η^2^ = 0.12].

**Conclusions:**

The moderate-intensity comprehensive exercise was confirmed as a safe and convenient exercise program. Although a load of training intensity is not sufficient to improve the gait speed, this exercise protocol is promising in delaying overall results in community-dwelling sarcopenia elderly females and contributes to the improvement of muscle mass, handgrip strength, and 5TCS.

## 1 Introduction

Sarcopenia, derived from the Greek (Greek ‘sarx’ or flesh + ‘penia’ or loss), was first proposed by Rosenberg in 1989 [1,2]. This age-related decrease in muscle mass can lead to low muscle strength and flexibility, and easy fatigue. Since 2016, this age-related muscle wasting has been recorded as sarcopenia, as a disease in ICD-10, and people's awareness of sarcopenia has rapidly increased. According to the EWGSOP guidelines, sarcopenia will affect >200 million in the next 30 years [3]. It is estimated that sarcopenia will become one of the essential factors threatening human health and social development with aging. In 2018, the European Working Group on Sarcopenia in Older People (EWGSOP), which summarized clinical practice and scientific research over the past decade, update the expert consensus. The consensus proposes that low muscle strength is an important feature of sarcopenia, and sarcopenia can be diagnosed by testing muscle mass and that low physical performance is considered a manifestation of severe sarcopenia [4].

As a progressive and skeletal muscle disease, sarcopenia is associated with an increase in negative consequences such as falls, disability, and lower quality of life in the elderly. The importance of preventing and managing sarcopenia is self-evident. Until now, there are no approved drugs for the treatment of sarcopenia, nutritional supplements, and exercise have received attention as a possible non-drug treatment for the prevention or treatment of sarcopenia. Protein intake is an important stimulant of muscle synthesis. However, studies showed that 15–38% of older men and 27–41% of older women consume less than the recommended daily allowance of protein [5]. Moreover, although leucine can offset the muscle loss caused by aging, and exercise can have a synergistic effect with the supplement of amino acids, the problem of a reasonable diet and healthy diet in the elderly is still a difficult problem in the health management of the elderly. As a commonly recommended prevention and treatment, exercise plays an important role in improving muscle mass and muscle strength in the elderly. Even though scientific studies have shown the beneficial effects of exercise on the prevention and treatment of sarcopenia in the elderly, the elderly with sarcopenia do not only experience a loss of strength, but also a loss of functional status, movement, flexibility, or balance [6]. These people are at increased risk of falls and fractures [7], which can lead to higher mortality [8].

It is urgent and necessary to pay attention to the improvement of physical performance in the elderly and delay the progression to severe sarcopenia. There are inconsistencies in the effectiveness of exercise interventions in improving physical performance in the elderly with sarcopenia. Kim [9] and Seo [10] proved that exercise could significantly improve gait speed, while Tsekoura [11] and Zhu [12] showed no changes in gait speed. More studies are needed to demonstrate its effect on improving muscle function in the elderly. As known, gait speed is also widely used as a predictor of physical performance in sarcopenia. Therefore, this study aims to set gait speed as the primary physical performance outcome to explore the effect of an 8-week exercise intervention on the improvement of physical performance in elderly with sarcopenia. Second, we also need to verify the impact of exercise on muscle strength and muscle mass in the elderly with sarcopenia.

## 2 Method

### 2.1 Study design

A prospective, dual-arm parallel design, community-based study was conducted. This study gained approval from the Institutional Review Board of the University of Ulsan (1040968-A-2022-005) and Henan University of Science and Technology (2022–0180).

The participants of this study were assigned to an experimental group (EG) receiving an eight-week moderate-intensity comprehensive exercise and a control group (CG), which was highlighted not to receive any exercise or nutrition program but only health education for 4 times. In accordance with the ethical standards of the Declaration of Helsinki, all participants were presented the detailed contents of the exercise intervention and then signed informed consent.

### 2.2 Participants

The subjects were recruited from the District of Jian Xi, Luoyang by posters, flyers, and WeChat. In accordance with the AWGS 2019 consensus, sarcopenia-associated parameters of 96 participants were examined to illustrate whether they conformed to the sarcopenia diagnosis criteria. The additional inclusion criteria comprised (a) participants aged ≥60 years, (b) independence ability (i.e., with an ability to finish TUG or 5TCS themselves), (c) no experience in a supervised exercise for three months prior to research. Any of the settings were excluded, comprising (a) diabetes mellitus, (b) cardiovascular diseases, uncontrolled hypertension (>140/90 mmHg), (c) gallstones, (d) kidney stone, (e) infectious diseases, (f) upper/lower extremity fracture, (g) knee or hip prosthesis, (h) pacemaker, (i) musculoskeletal disorders, (j) psychiatric disorders, (k) epilepsy, (l) any medical treatment that affects sarcopenia outcomes, (m) cognitive impairment (Mini-Mental State Examination, MMSE score <23); (o) physical dysfunctions.

### 2.3 Sample size

We performed an a prior power analysis using G*Power 3.1 [13] for the sarcopenia index based on previous research [14], This analysis indicated that a sample size of 33 participants would be required to provide 80% power at an a-level of 0.05. According, it needs at least 42 participants if conducted with a statistical power (1-β) of 0.90, and α value of 0.05. Lastly, we included about 60 elderly females with sarcopenia (the study was conducted during the midst of a Covid-19 pandemic, so we set a 30% dropout) from a community dwelling.

### 2.4 Randomization

The participants were asked to offer written informed consent after understanding the objective, rights, and potential risks. One trained researcher (CYZ) estimated the participants’ baseline characteristics. Then, an independent researcher (SSH) used a computer-generated list of random numbers [15] to randomly allocate 60 participants into the experiment group (EG = 30) or a control group (CG = 30). The allocation sequence will be covered in sealed envelopes, which will not be unfolded until the baseline assessment is completed. Two exercise specialists (CBY, CZL) would be concealed before the intervention, but they could not be blinded during the exercise period, because of the character of the intervention. After an eight-week intervention, researcher CYZ estimated the sarcopenia-associated parameters again, and professional (DH) would perform the statistical analysis. Both of them (CYZ, DH) will be blinded to the group assignment of study participants.

### 2.5 Interventions

This comprehensive program takes on critical significance in muscle function, and physical performance (e.g., the trunk, upper, and lower muscle groups training). The respective group was administrated and guided by trained exercise specialists to ensure adherence to this protocol. The respective exercise session comprised nearly 60 min, three times per week for eight weeks, mainly including a 5-min warming-up, followed by a training exercise part of 45–50 min (combined simplified 24-form Tai Chi and progressive resistance training) in a broad and well-cool indoor activity room with a group-based setting, and then a 5-min cool-down part. The exercise program was conducted at a moderate intensity roughly 12–14 on the Borg rate of perceived exertion scale (Borg RPE). The experiment group was part into four subgroups to guarantee participants the appropriate and convenient instruction. All the participants practiced within their allocated subgroup.

#### 2.5.1 Simplified 24-form Tai Chi exercise

Tai Chi might be benefit for improving balance, reducing falls and injury-related falls in older people [16], and improve physical function performance and disease-specific adverse outcomes among adults with diverse chronic illnesses [17]. The simplified 24-form Tai Chi was exercised 3 times a week, for a total of 24 sessions. 2 sets of simplified 24-form Tai Chi would be practiced in each session. The training program was guided by a Tai Chi master with over 15 years of teaching experience. All the participants learned the simplified 24-form Tai Chi for two weeks before the intervention, in order to make sure they could able to complete the simplified 24-form Tai Chi program in every session during the eight weeks.

#### 2.5.2 Progressive resistance exercise

The progressive resistance exercise program was performed using elastic bands. Different colors of the above-mentioned elastic bands represented different resistance levels, with nearly a 20% resistance intensity difference between the respective color (yellow, red, green, blue, black, or silver). To be specific, two days before the exercise intervention and one day before the fifth week the tests were performed to predict the respective participant's one-repetition maximum (1RM) in first-fourth and fifth-eighth weeks through a submaximal 60–80% RM test. In the first-fourth weeks, the exercise protocol was the same, the intensity varied every two weeks, as well as in the fifth-eighth weeks. However, some difference was identified between the first-fourth and fifth-eighth weeks, in which the training content and the repetition/set were modified. Furthermore, since the participants were the elderly, nearly a 45-s to 90-s rest was provided prior to the next part of the content.

In terms of the actual sessions, participants were instructed and practiced four movements. The participants were guided to practice the respective movement at 40% of 1RM intensity using a yellow TheraBand initially first, and then the 1RM intensity was progressively increased to 60–80% with the adjustment to two-three sets of 8–12 repetitions. To facilitate the development of muscle function in the upper and lower body, participants were highlighted to do the respective concentric movement ‘as soon as possible’ and perform the eccentric movement in a relaxed and controlled status. Higher-intensity resistance was enhanced when the participants showed the capability of maintaining their RPE to a “somewhat hard” grade. However, if participants could not accept the next progressive intensity (i.e., from green to blue), the previous resistance intensity of the elastic band was maintained for an additional session until the participants could receive the next elastic band intensity color. The planning and implementation of the protocol were in accordance with the guidelines supported by the American College of Sports Medicine.

#### 2.5.3 Control group

Participants in the CG will not be subjected to any recommendations about altering their daily diet or lifestyle during the eight weeks. They will receive calls or interviews once a week, and any health problem information was written down by a trained researcher. Meanwhile, health education was prescribed to them per two weeks. The topics of health education included primary healthcare skills, cognitive ability, etc., but were not shared with exercise or nutrition. CG subjects also received the same pre- and post-test as EG participants.

### 2.6 Outcome measurement

#### 2.6.1 Body composition assessment

Height and weight were estimated by a height and weight machine (SH-500, China) to the nearest 0.1 cm and 0.1 kg, respectively, without shoes. SMM and ASM were estimated according to BIA [18], and their reliability was previously validated [19]. The participants were asked to shift any metal they carried before the test, then stand on insulating malposition, spread their legs, and arms hung down at a 15-degree to the trunk. Then, the researcher put the electrode device on the participants’ arms and legs for in-depth assessment. Following the AWGS 2019 consensus, muscle mass (SMI) was assessed with ASM adjusted with height [18], and low muscle mass was considered a cut-off of less than 5.7 kg/m^2^ in females.

#### 2.6.2 Handgrip strength

Handgrip strength is considered a feasible and convenient measure of measuring muscle strength for use in hospital services, clinical research, and primary health care settings [20]. AWGS 2019 retains the recommendation to use handgrip strength to forecast skeletal muscle strength [19]. According to the handgrip strength measurement protocol, the participants were asked to hold the dynamometer (Jamar, Chicago, USA) in a sitting position, with 90° elbow flexion with 20–30° to the body. Subsequently, the dynamometer was grasped on the dominant hand two times, each for nearly three to 5 s, and the maximum reading performance was recorded. The cut-off threshold for handgrip strength was less than 18 kg for women in accordance with the AWGS 2019 consensus.

#### 2.6.3 Physical performance

AWGS 2019 recommends that either one of the SPPB, gait speed (6-m walk) or 5-time chair stand test lower than the cut-off threshold could be defined as low physical performance [19]. In this study, gait speed and 5TCS were examined, each of which represents the physical performance status. The participants were requested to walk 6 m at a preferred speed, without deceleration, and calculate the average result of at least 2 trials as the tested speed. As mentioned, AWGS 2019 defined low physical performance as a 6-m walk <1.0 m/s [19]. Moreover, 5TCS was considered a replacement measurement standard in sarcopenia diagnosis. The participants should cross their arms to their chest and sit down in a chair, and then begin to stand up and sit down five times as soon as possible. The cut-off value was defined as more than 12 s, i.e., low physical performance.

### 2.7 Statistical analysis

The selected recording data were examined using SPSS 26.0 (IBM SPSS Statistics for Windows, USA) in accordance with the following steps.

1) All data were checked for outlier's presence, normal distributions (Kolmogorov–Smirnov test), and homogeneity of variance (Levene's test).
2) Descriptive statistics were computed for all characteristic variables at baseline. Moreover, pre-intervention differences in the variables between groups were examined to test whether any clinical or anthropometric items could be added as a covariate in the analysis of variance (ANOVA).
3) The interactions between measured variables were calculated through a two-way repeated ANOVA measures of the time factor to analysis the EG and CG groups and test the effects of the 8-week exercise on each variable. The Bonferroni post hoc was performed when significance was found.
4) α = 0.05 indicated a difference with statistical significance. Data are expressed as mean ± SD unless otherwise stated.

## 3 Results

**Fig. 1:**
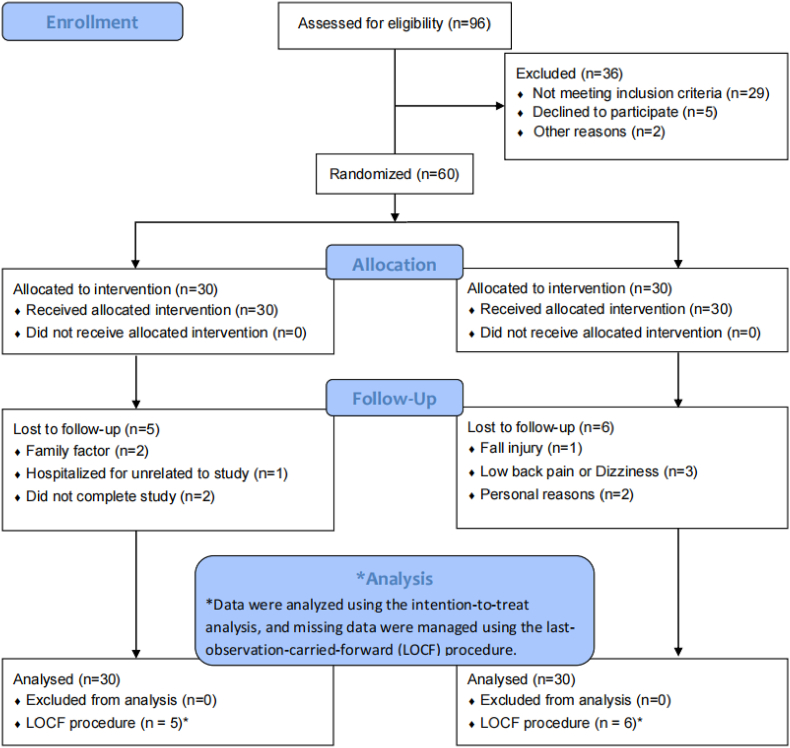
Flow diagram of sarcopenia participants' selection and allocation.

A total of 60 eligible female participants were recruited. Participants were randomly assigned to an EG (n = 30) or a CG (n = 30). 11 participants withdrew from the study for personal reasons, and 49 participants (EG = 25, CG = 24) completed the exercise intervention session. The mean rate of attendance reached 81% in the EG. No severe adverse events or health problems were identified during the intervention. Details as displayed in the flowchart in Fig. 1.

There were no significant differences (p > 0.05) in any of the variables were found between the experiment group and control groups. Baseline values of the characteristics are reported in Table 1.

**Table 1:** Participant characteristics at baseline (M±SD).

Variables	EG	CG
Age (year)	65.68 ± 2.5	65.21 ± 2.6
Height (cm)	161.7 ± 4.1	162.2 ± 5.2
Weight (kg)	60.89 ± 3.41	60.49 ± 4.0
ASM (kg)	13.74 ± 0.68	13.71 ± 0.76
SMM (kg)	19.10 ± 0.88	19.02 ± 0.66
SMI (kg/m^2^)	5.26 ± 0.16	5.21 ± 0.12
Handgrip strength (kg)	15.94 ± 0.90	15.89 ± 0.98
Gait speed (m/s)	1.20 ± 0.11	1.18 ± 0.10
5TCS (s)	11.27 ± 0.61	11.40 ± 0.55

Note: Average (mean ± SD) values of age, height, weight, appendicular skeletal muscle mass (ASM), skeletal muscle mass (SMM), ASM/Height^2^ (SMI), and 5-time chair stand (5TCS) at baseline for the EG and CG.

### 3.1 Effect of exercise intervention on body composition

As shown in Table 2, the two-way interaction of groups and time were significant for ASM [F (1,47) = 6.25, η^2^ = 0.12)], SMI [F (1,47) = 6.77, η^2^ = 0.13]. Significant interactions were examined with tests of simple effects. The simple effect test showed that the EG was significantly higher in sarcopenia index at W8 than the CG, with a 0.15 kg/m^2^ improvement (p < 0.01). In addition, the EG reported a significantly higher ASM and sarcopenia index at W8 than at W0. There was no significantly difference recorded in the CG at W8 than at W0.

**Table 2:** Changes (mean ± SD) in muscle mass (body composition, sarcopenia index), muscle strength (handgrip strength), and physical performance (gait speed, 5-time chair stand test) before (baseline), after 8-week comprehensive exercise intervention for the EG and CG.

	Experimental group (N = 25)		Control group (N = 24)	
	Baseline	Week 8	Baseline	Week 8
ASM (kg)	13.74 ± 0.68	14.00 ± 0.69^#^	13.71 ± 0.76	13.69 ± 0.86
SMM (kg)	19.10 ± 0.88	19.28 ± 0.82	19.02 ± 0.66	18.92 ± 0.83
SMI (kg/m^2^)	5.26 ± 0.16	5.36 ± 0.21 *^,#^	5.21 ± 0.12	5.20 ± 0.13
Handgrip strength (kg)	15.94 ± 0.90	16.74 ± 0.86 *^,#^	15.89 ± 0.98	15.86 ± 0.96
Gait speed (s)	1.20 ± 0.11	1.25 ± 0.12^#^	1.18 ± 0.10	1.18 ± 0.12
5TCS (s)	11.27 ± 0.61	11.00 ± 0.39 *^,φ^	11.40 ± 0.55	11.42 ± 0.39

ASM = appendicular skeletal muscle mass, SMM = skeletal muscle mass, SMI = ASM/height.^2^.

⁎ Denotes that the EG exhibited significantly difference than that of the CG during the same period (p < 0.05).

# The number is significantly higher than that at Baseline (p < 0.05).

φ The number is significantly lower than that at Baseline (p < 0.05).

### 3.2 Effect of exercise intervention on muscle strength

The two-way interaction of groups and time were significant for handgrip strength [F (1,47) = 6.80, η^2^ = 0.13]. Significant interactions were examined with tests of simple effects. The simple effect test showed that the EG was significantly higher effect (F = 4.9, η^2^ = 0.1) at W8 compared to the CG. In addition, at W8, the EG reported a significantly 0.88 kg (p < 0.01) higher recording than at W0. On the other hand, the CG were no significantly difference than at W0, see Table 2.

### 3.3 Effect of exercise intervention on physical performance

At week 8, The EG showed a significant difference in gait speed, with a 0.05 m/s (p < 0.01) improvement, and 5TCS was reduced by 0.27 s (p < 0.01) to baseline. The two-way interaction of groups and time were significant for 5-time chair stand [F (1, 47) = 6.35, η^2^ = 0.12]. Significant interactions were examined with tests of simple effects. The simple effect test showed that the EG and the CG had a 0.42s (p < 0.01) difference between groups at W8. No statistical difference was identified in the gait speed and 5TCS at W8 in the CG, see Table 2.

## 4 Discussion

This study confirmed the impact of an 8- week moderate-intensity comprehensive exercise protocol (combined Tai Chi and TheraBand progressive resistance training) on sarcopenia-associated parameters in community-dwelling elderly female sarcopenia. The primary finding is the eight-week moderate-intensity comprehensive exercise program improved 5TCS in elderly females with sarcopenia, but there is no significant difference recorded in the gait speed. The second finding is muscle mass, handgrip strength had a positive change after this eight-week comprehensive exercise intervention. Thus, the sarcopenia-associated outcomes (without gait speed) in elderly females with sarcopenia occurred beneficial change after eight weeks of the comprehensive exercise protocol.

The present study presents evidence to support the benefits of exercise on muscle mass (ASM/height^2^). Generally speaking, skeletal muscle mass represents a crucial of strength, endurance, and physical performance. A previous 24-week combined resistance exercise and Yi Jin Jing had a positive effect on increasing skeletal muscle mass and delaying sarcopenia in the elderly [21], which confirmed our results findings.

Previous articles have proved the relationship between Tai Chi and muscle strength in the elderly [22,23], but most of these articles focused on lower body strength [24], balance [25], and cardiorespiratory [26]. Our finding results indicated that the moderate-intensity comprehensive exercise program conducted optimistic functions on muscle strength. These useful results could show clinicians to systematically serve their patients, especially sarcopenia.

At present, an outstanding decrease in natural capacity in the elderly is shown by the overall performance of universal issues, such as feeling hard to walk at normal speed, continuous muscle wasting, and muscle strength and function impairments. De Carvalho [27] declared the necessity for earlier diagnosis and assessment of physical performance in daily healthcare to gain better prevent physical function decline (clinically expressed as mobility impairments). The ACSM recognized resistance training as essential for the elderly’ physical performance [28]. The current exercise program significantly strengthens physical performance (5TCS) in the sarcopenia elderly. Lin [29] also demonstrated that a comprehensive exercise program could promote community-dwelling old adults’ physical function.

Nevertheless, the comprehensive exercise program was no significant effect on improving the usual gait speed in elderly females with sarcopenia in the present study, which was also summarized in earlier articles [12,30,31]. Uematsu [32] provided that hip extensors and ankle plantar flexors became the only significant predictors of self-selected and maximal gait speed. Lim [33] previously evidenced that physical exercise interventions were more effective within those at-risk older adults for increasing maximal gait speed, but not preferred gait speed. A possible explanation could be that within the exercise protocol, participants did exercises such as leg press and extension, low limb squat, and raise that may have had a greater impact on 5TCS capacity but had no significant improvement to mobility such as self-selected speed. Accordingly, more targeted exercise protocols might be needed and designed for women to improve gait speed. These are important considerations for future exercise delivery in the community-dwelling context.

In our study, combined Tai Chi and TheraBand progressive resistance exercise programs had a significant effect on sarcopenia-associated outcomes. The prescription involved not only upper and lower strength training, but also waist, knee bending, waist stretching, and balance. These present findings suggested this exercise protocol could be an effective way to promote safer locomotion and improve sarcopenia-associated outcomes in community-dwelling elderly females. Thus, we suggest older people take part in this comprehensive exercise program. Moreover, the elderly could adjust the pull force of TheraBand to improve exercise intensity, as well as keep body gravity at a lower position when practicing this comprehensive exercise program, which might enhance more benefits to overcome physical performance change.

Several limitations remained in this study. First, it became more difficult to take part in group-based exercise activities under the effect of the COVID-19 pandemic, such that the diversity in subject recruitment was subjected to a shortage. Second, a more compared group should be set (e.g., introducing the Tai Chi group and the resistance training group to a more effective horizontal comparison for obtaining more comprehensive and accurate results). Furthermore, more intensity subgroups should be separated to determine the optimal frequency/time. Lastly, mental health has taken rising significance in the sarcopenia elderly, and more studies should be conducted to analyze the effects of exercise on their satisfaction, self-efficacy, or quality of life.

## 5 Conclusions

The sarcopenia elderly should participate in an appropriate type of exercise program during non-pharmacological interventions to relieve or transform their sarcopenia status for better health and quality of life. The present moderate-intensity comprehensive exercise refers to a combination of aerobic, balance (simple 24-form Tai Chi) and resistance, and flexibility exercise (TheraBand exercise) in one exercise program strategy, i.e., a safe and convenient exercise program.

Although a load of training intensity is not sufficient to improve the gait speed, this exercise protocol is promising in delaying overall results in community-dwelling sarcopenia elderly females and contributes to the improvement of muscle mass, handgrip strength, and 5TCS. Thus, community staff or sports instructors are recommended to receive the training for implementing this comprehensive exercise protocol program for the community-dwelling elderly with sarcopenia.

## Author contribution statement

Chen Bo-yuan: Conceived and designed the experiments; Performed the experiments; Wrote the paper.

Shin So-hee: Conceived and designed the experiments; Performed the experiments; Analyzed and interpreted the data; Wrote the paper.

Jie Chun-yang: Contributed reagents, materials, analysis tools or data; Wrote the paper.

Chen Yuan-zhe, Chang Zhi-liang and Ding Hui: Performed the experiments; Analyzed and interpreted the data.

Yang Hong: Performed the experiments.

## Data availability statement

Data included in article/supp. Material/referenced in article.

## Additional information

No additional information is available for this paper.

## Declaration of competing interest

The authors declare that they have no known competing financial interests or personal relationships that could have appeared to influence the work reported in this paper.

## Figures and Tables

**Table:** 

	Movement		Intensity	Repetition	Set	Targeted muscle group	Duration (min)
A: warm-up	1. Mobility exercise of the neck, upper limbs, and back		Low	8	1	Whole-body	5
	2. Global flexion-extension of the lower limb						
B: Exercise	1. TaiChi		Moderate	1	2	Lower limb balance and stability	20
	2. Resistance Exercise	biceps curl, horizontal abduction, flexion and extension	60–80% RM; Borg RPE 12-14	10(Weeks 1–2) 12(Weeks 3–4) 8(Weeks 5–6) 10(Weeks 7–8)	2(Weeks 1–4) 3(Weeks 5–8)	Upper limb	20–30
		torso rotation, rotate push up & down				Trunk and Abdominal	
		hip adduction & abduction, hip flexion, calf press, external rotation				Lower limb	
C: Cool down	flexible stretch		Low	8	1	Whole-body	5
